# An Exploratory Randomized Controlled Trial of 0.9% Saline Versus Lactated Ringer's Solution on Intraoperative Metabolism Among Patients Undergoing Lumbar Spinal Surgery

**DOI:** 10.7150/ijms.128640

**Published:** 2026-02-11

**Authors:** Fon-Yih Tsuang, Yi-Luen Wu, Kuang-Cheng Chan, Chen-Tse Lee, Chun-Yu Wu

**Affiliations:** 1 Department of Neurosurgery, National Taiwan University Hospital, Taipei, Taiwan.; 2 Spine Tumor Center, National Taiwan University Hospital, Taipei, Taiwan.; 3 Department of Anesthesiology, National Taiwan University Hospital, Taipei, Taiwan.; 4 Department of Anesthesiology, National Taiwan University Hospital Hsinchu Branch, Hsinchu city, Taiwan.; 5 National Taiwan University College of Medicine, Taipei, Taiwan.

**Keywords:** normal saline, balanced crystalloid, metabolomics, spine surgery

## Abstract

**Introduction:**

Intravenous crystalloid fluid infusion is a mandatory nutritional intervention received by surgical patients. Crystalloids vary in the pH value and electrolyte balance when administered; these effects directly alter plasma and urine compositions and can considerably affect the patient's intraoperative metabolism.

**Methods:**

This randomized controlled study compared 0.9% saline solution and lactated Ringer's solution in terms of intraoperative metabolism among 56 patients undergoing lumbar spinal surgery. Blood and urine samples were obtained before and after surgery for arterial blood gas analysis and untargeted metabolomic analysis using liquid chromatography-mass spectrometry.

**Results:**

Patients receiving 0.9% saline developed hyperchloremic acidosis and exhibited higher postoperative plasma concentrations of sodium (interaction P = 0.008) and glucose (interaction P = 0.034). They also required higher intraoperative norepinephrine doses (18 [0-43] μg vs. 0 [0-5] μg; P < 0.001) compared with patients who received lactated Ringer's solution. Significant intraoperative changes were noted in 27 plasma metabolites and 32 urinary metabolites related to stress responses and catabolic pathways. Patients receiving lactated Ringer's solution exhibited favorable metabolism in the blood. This was indicated by attenuated cortisol concentrations (interaction *P* = .051); significantly higher oxaloacetate concentrations (interaction *P* = .015), which may indicate less intraoperative gluconeogenesis (interaction *P* = .015); lower leucine degradation metabolite concentration, namely hydroxyisocaproic acid (interaction *P* = .055); and an attenuated decline in anti-inflammatory phospholipid breakdown metabolite, namely 15-ketoeicosatetraenoic acid (interaction *P* = .063). By contrast, patients receiving 0.9% saline solution exhibited unfavorable metabolism in urine indicated by reduced excretion of citric acid and creatine, which correlated with reduced glomerular filtration rates.

**Conclusions:**

The administration of lactated Ringer's solution may facilitate more favorable intraoperative metabolic profiles than the administration of 0.9% saline solution during lumbar spinal surgery.

## Introduction

Intravenous crystalloid fluid infusion is the most common intervention administrated to surgical patients 1 and is increasingly regarded as a pharmacological prescription, given that fluid choice may influence postoperative outcomes 2. Two classes of crystalloids are the most commonly prescribed by clinicians, namely saline (0.9% sodium chloride) and balanced crystalloids, among which lactated Ringer's solution is commonly prescribed. Crystalloids vary in the plasma pH value, states of base excess, and electrolyte balance when administered 3; these effects directly alter plasma and urine compositions and can considerably affect the patient's intraoperative metabolism. Surgical stress threatens metabolic homeostasis by facilitating cortisol secretion, which can induce hyperglycemias (gluconeogenesis), protein catabolism, and lipid oxidation; these perturbations may trigger postoperative organ injuries 4. Therefore, knowledge regarding the mechanisms through which different types of intravenous crystalloid solutions affect intraoperative metabolism is key to providing individualized intervention in the era of precision perioperative medicine 5, 6. In patients who are susceptible to the biochemical properties of crystalloid solutions—such as the risk of hyperchloremic metabolic acidosis with 0.9% saline—the use of balanced crystalloids may result in more favorable postoperative outcomes. For instance, balanced crystalloid intravenous fluid therapy has been shown to reduce the incidence of delayed graft function and maintain more favorable serum chemistry profiles compared with 0.9% saline in kidney transplantation recipients 7. Therefore, it is of considerable interest to conduct an exploratory trial to further investigate the potential metabolic effects of these two types of crystalloids in surgical populations.

The recent development of metabolomics enables quantification using small amounts of biofluids, such as blood and urine, and of low molecular weight metabolites, all of which represent the endpoints of the omics cascade 8. Metabolomics involves the detection of the relevant cascade of intraoperative phenotypes, and these novel analytic techniques have thus been increasingly applied to the delivery of precision perioperative medicine to patients undergoing surgery 6, 8. Intravenous fluids are of particular interest because crystalloids directly affect the composition of biofluids. The type of surgery also significantly affects the patient's intraoperative metabolic profile. Lumbar spinal surgery may be useful for investigations on the choice of crystalloid solution for several reasons. First, the use of lumbar spinal surgery has increased considerably worldwide in the last 20 years which presents a relevant global burden 9. Second, fluid management has been recognized as an important component of lumbar spinal surgery because the prone positioning and blood loss incurred in lumbar spinal surgery predispose patients to high intravenous fluid requirements 10. Despite the recent study already reported that surgery imposes broad-scale early and measurable metabolic changes 11, the degree to which these changes are influenced by the specific choice of intraoperative crystalloid remains poorly understood. Most existing metabolomic studies in the surgical setting have been observational or focused primarily on diagnostic outcomes. This study addresses this gap by utilizing untargeted metabolomics within a randomized controlled trial framework to compare the metabolic signatures of 0.9% saline and lactated Ringer's solution. By focusing on a homogeneous cohort of patients undergoing lumbar spinal surgery, we aim to provide novel mechanistic insights into how crystalloid choice influences the intraoperative stress response and renal metabolic profile, thereby contributing to the development of precision perioperative fluid management.

## Materials and Methods

### Study design and participants

This study was designed as an exploratory randomized controlled trial. While its primary goal was the discovery of intraoperative metabolic alterations rather than testing a specific metabolite-driven hypothesis, we maintained the rigorous standards of a clinical trial, including randomization and blinding, to ensure data integrity. The protocol for this study was approved by the Research Ethics Committee of National Taiwan University Hospital (approval number: 201907091RINA; date of approval: 18 April, 2019) and was registered at clinicaltrials.gov prior to patient enrolment (NCT04137042; date of clinical trial initiation: 18 November 2019; principal investigator: Chun-Yu Wu; date of registration: 23 October, 2019; https://clinicaltrials.gov/ct2/show/NCT04137042). This study was performed in accordance with the Declaration of Helsinki and the manuscript was prepared following the Consolidated Standards of Reporting Trials (CONSORT) 2025 checklist.

Due to the association between age and metabolism, we included only middle-aged patients between 40 and 65 years, as middle-aged patients account for the majority of surgical patients worldwide 12. In addition, inclusion of these relatively healthier population may help reducing age-related metabolic variability to facilitate the study's interests on the selection crystalloid solution for surgical metabolism. These patients received major lumbar spinal surgery with an expected anesthetic time longer than 2 h. Patients were excluded if they received diuretic before surgery; had an ASA physical status score of 3 or more; had preoperative organ dysfunction—including impaired liver function (e.g., aspartate aminotransferase or alanine aminotransferase > 100 u·l^-1^), liver cirrhosis ≥ Child B class, impaired renal function (with an estimated glomerular filtration rate < 60 ml·min^-1^·1.73 m^-2^), or cardiac dysfunction (e.g., heart failure ≥ New York Heart Association class II)—coronary artery disease; chronic obstructive pulmonary disease; an ongoing infectious disease; morbid obesity; or a medication regime that significantly interfered with liver, renal, or carbohydrate metabolism (including medications such as insulin, statins, or digoxin). As this trial was conducted during the SARS-CoV-2 pandemic, patients scheduled for elective surgery underwent polymerase chain reaction testing the day before surgery, and only those with negative results proceeded to surgery.

Written informed consent was obtained from each patient the day before surgery. Patients were blindly allocated to receive either the 0.9% saline (NS group) or the lactated Ringer's solution (LR group) at a 1:1 ratio according to a predefined block randomization list. Patients were blinded to group allocation. Although the attending anesthesiologists could not be blinded due to the distinct packaging of the crystalloid solutions, both the researchers performing the metabolomic analyses and the data analysts remained blinded to group assignments.

### Perioperative management

Each patient fasted from solid foods for 6 h and was encouraged to consume clear liquids and oral carbohydrates 2 h before surgery with preoperative crystalloid administration 1. Different operating theatre environments may affect intraoperative metabolism differently 6; thus, this study was conducted in a single operating theatre. Each patient underwent general anesthesia induction comprising fentanyl (2-3 μg·kg^-1^), propofol (1.5-2.5 mg·kg^-1^), and cisatracurium (0.2 mg·kg^-1^). Anesthesia was maintained through sevoflurane at a bispectral index of 40-60. Each patient was ventilated in pressure-controlled volume-guaranteed mode with a tidal volume of 6-8 ml·kg of ideal body weight and a positive end-expiratory pressure of 5 cmH_2_O. The fraction of inspired oxygen was maintained at 50%, and the respiratory rate was adjusted to maintain an end-tidal CO_2_ level of 35-45 mmHg. Body temperature was maintained at >36.5 °C by using a Bair Hugger temperature management system (3M, St. Paul, MN, USA). Each monitor was installed before anesthesia induction. The standard monitors applied in this study comprised a Philips IntelliVue MP70 monitor (Philips Medical Systems, Suresnes, France) and a Bispectral Index Quatro sensor (Covidien LLC, Mansfield, MA, USA) connected to a Philips BIS M1034 module (Philips Medical Systems, Germany). Each patient in both the groups received background infusion of the allocated crystalloid solution at 2 mL/kg/h. In addition, a radial arterial catheter was placed to ensure that pulse pressure variation was >13% 13 using fluid challenge with the predefined crystalloid solution. Mean arterial pressure was maintained at ≥65 mmHg 14 using intravenous norepinephrine titration.

### Biofluid sampling

Biofluids including 10 ml blood and 10 ml urine were collected at two time points via radial arterial and urinary catheters placed immediately after anesthesia; preoperative samples were obtained immediately after anesthesia, and postoperative samples were obtained after skin closure. The postoperative urinary sample contained no intraoperative urine. Biofluid samples were collected into heparinized tubes, plasma was immediately separated by centrifugation at 3000 rpm for 10 min, and both biofluid samples were stored at -80 °C until metabolomic analysis. In addition, arterial blood gas analysis was measured at the same time points as the biofluid sampling for metabolomic analysis.

### Untargeted metabolomic profiling

The samples were thawed at 4 °C on ice immediately prior to analysis. A 100-μl sample was transferred to an eppendorf tube, and 300-μl and 400-μl samples of extract solution (methanol/acetonitrile: methanol = 1:1) were added to the serum and urine samples, respectively. After 30 s of vortexing, the samples were homogenized for 5 min and sonicated for 10 min on ice. The samples were then incubated for 1 h at -20 °C and centrifuged at 12 000 rpm for 15 min at 4 °C. The resulting supernatant was transferred to a fresh glass vial for further analysis. A quality control sample was prepared by mixing an equal aliquot of the supernatants from all of the samples 15.

A 10-μl sample was injected into a vanquish focused ultra-high-performance liquid chromatography (LC) system coupled with an Orbitrap Elite Mass Spectrometry (Thermo Fisher Scientific, USA) using electrospray ionization (ESI). Chromatography parameters were configured as follows. A 2.1 × 100 mm ACQUITY BEH 1.7 μm C18 column (Waters, USA) was used. The column oven temperature was 40 °C. The binary mobile phase used deionized water containing 0.1% formic acid as solvent A and LC-mass spectrometry (MS)-grade acetonitrile with 0.1% formic acid as solvent B. The flow rate was 0.25 ml·min^-1^ with a linear gradient elution for 15 min. For the first minute, the percentage of solvent B was held at 0%, linearly increased to 100% for the next 7 min, kept constant for 3 min, then finally returned to 0% in 1 min. To avoid any carry over effect, blank injection was performed after every sample injection, and one quality control injection was performed after every five sample injections for peak area normalization 15. MS data were collected in positive mode with a default data-dependent acquisition method. One MS full scan was performed in profile mode at 60 000 resolutions, followed by 10 data-dependent MS^2^ scans at 15 000 resolutions. The MS scan range was 70-1000 m·z^-1^. The normalized collision energy was 25. The spray voltage was 3.5 kV. The capillary temperature was 280 °C. The sheath gas was 30 arbitrary units and the aux gas was 5 arbitrary units.

### Data preprocessing and annotation

Raw data files were converted to mzML format using ProteoWizard and processed using the R package XCMS (version 3.2). After preprocessing, a data matrix composed of the mass-to-charge ratio (m/z), retention time, and peak intensity values was generated. Subsequently, each sample peak was normalized and quantified using the area under the curve. Subsequently, each peak was annotated using an in-house MS2 database. The scoring cutoff for annotation was set at 0.5 16. For multivariate analysis, outliers (all values that were lower or higher than 1.5 times the interquartile range from the 25% and 75% quantiles) were eliminated from the raw dataset. Missing values of variables, for groups in which more than half of all samples were missing, were filled with half of the minimum intensity value 17. Furthermore, the normalized data were used to perform multivariate statistical analysis, namely the orthogonal projection to latent structures-discriminant analysis (OPLS-DA) and to obtain variable importance in projection values, which was set to 0.5 for the lowest cutoff relevance for the OPLS-DA model 18, to evaluate important contributions of the metabolites to classification. This validated multivariable technique allows for the analysis of multiple analytes to differentiate the metabolic profiles of different groups. The R^2^Y (a measure of goodness of fit) and Q^2^ (consistency on cross-validation) values were reported for each model.

### Statistical analysis

Data distributions were visually evaluated using histograms. Categorical variables are presented as the frequency (percentage) and compared using Fisher's exact test or the chi-square test as appropriate, reported as relative risk (95% CI). Continuous variables are presented as mean (standard deviation) or median (interquartile range). Balance in the baseline characteristics was assessed on the basis of standardized differences 19; a standardized difference of >0.52 was considered to indicate an imbalance between the groups.

Because metabolomic analysis is associated with multiple testing, we applied a permutation-based correction, which accounted for metabolite collinearity and prevented the tendency toward overconservative estimates in the conventional Bonferroni's method 20, 21. Peak postoperative metabolite MS values were compared with preoperative values using the paired *t* test or the Wilcoxon matched-pairs signed-rank test. The metabolites were considered statistically significant with a permutation corrected *P* < .05 16, 20. Data in the perioperative profile such as the baseline and intraoperative variables and hospital stay, were compared between the groups using Student's* t* test. Repeated measures analysis of variances was used to identify potential intraoperative metabolomic differences between the two crystalloid solutions. A group by time interaction effect with *P* < .10 indicated a statistical trend strongly toward significance 22 and this interaction effect reflects differential perioperative trajectories between crystalloid groups, rather than isolated postoperative differences. This is considered to be acceptable for the assessment of treatment-outcome relationships 23, 24. Heat maps of plasma and urinary metabolite levels were generated using hierarchical clustering based on Pearson's correlation coefficient.

Precise sample size estimation in metabolic phenotyping is difficult because the priori metabolic target is often absent. Previous studies have found that a minimum of 20 samples is sufficient to perform a robust power analysis 25, 26. Therefore, we planned to enrol 28 patients in each group (total 58 patients) with a power of 0.8, a two-sided type I error of 0.05, and consideration for potential attrition. All statistical analyses were performed on SAS (version 9.4; SAS Institute, Cary, NC, USA) with a two-tailed *P* value of <.05 indicating a significant difference.

## Results

**Figure 1:**
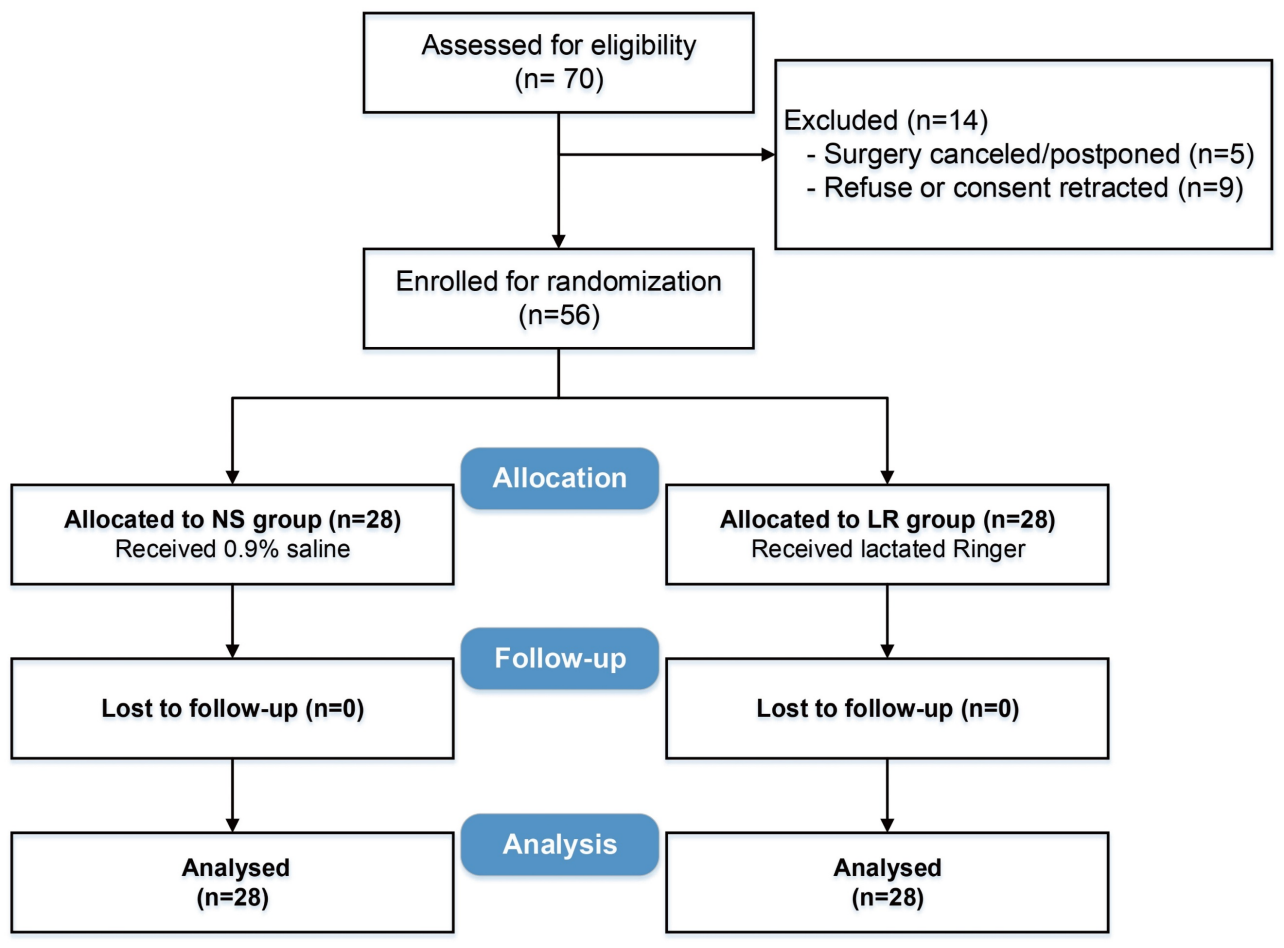
Study flow diagram.

Between November 2019 and May 2021, 70 patients were screened, of whom 56 were enrolled and randomized into two groups (Figure 1). The two groups were adequately balanced on baseline characteristics and surgery types, with all standardized differences within the predefined acceptable range (Table 1).

### Intraoperative profiles and postoperative outcomes

The two groups had comparable intraoperative profiles in terms of anesthetic time, blood loss, urine output, and infused intravenous fluid amount (1706 ± 522 and 1588 ± 511 ml for the NS and LR groups, respectively, *P* = .394, Table 3). By comparison, the median norepinephrine dose was 18 μg (IQR, 0-43 μg) in the NS group, which was significantly higher than that in the LR group at 0 μg (IQR, 0-5 μg; *P* < 0.001; Table 2). No diuretics were administered to any patient during surgery. With regard to postoperative outcomes, one patient in the NS group developed a wound infection, one patient in each group developed new neurological symptoms, two patients in the LR group developed transient arrhythmia, and one patient in the NS group had rebleeding that required transfusion. The incidences of complications were comparable between the two groups (P = .406), and both groups did not significantly differ in the length of hospital stay (5 [4-6.5] and 4 3-5 days in the NS group and LR group, respectively, P = .235, Table 2).

Table 3 summarized intraoperative arterial blood gas profiles of the two study groups. The two groups did not significantly differ in baseline arterial blood gas profile. However, patients receiving 0.9% saline solution had a higher prevalence of acidosis (interaction *P* = .034), hypernatremia (interaction *P* = .003), and hyperchloremia (interaction *P* = .034) and a higher plasma glucose elevation (interaction *P* = .034) after surgery. To facilitate clinical interpretation, we additionally reported absolute perioperative changes and the between-group difference in change (difference-in-differences, ΔΔ) with 95% confidence intervals. Compared with lactated Ringer's solution, 0.9% saline was associated with a greater decline in arterial pH (ΔΔ -0.024 pH units, 95% CI -0.047 to -0.002), and greater increases in sodium (ΔΔ +1.389 mmol/L, 95% CI 0.372 to 2.407), chloride (ΔΔ +1.607 mmol/L, 95% CI 0.582 to 2.633), and glucose (ΔΔ +11.179 mg/dL, 95% CI 0.495 to 21.862).

### Intraoperative metabolomic profiles

**Figure 2:**
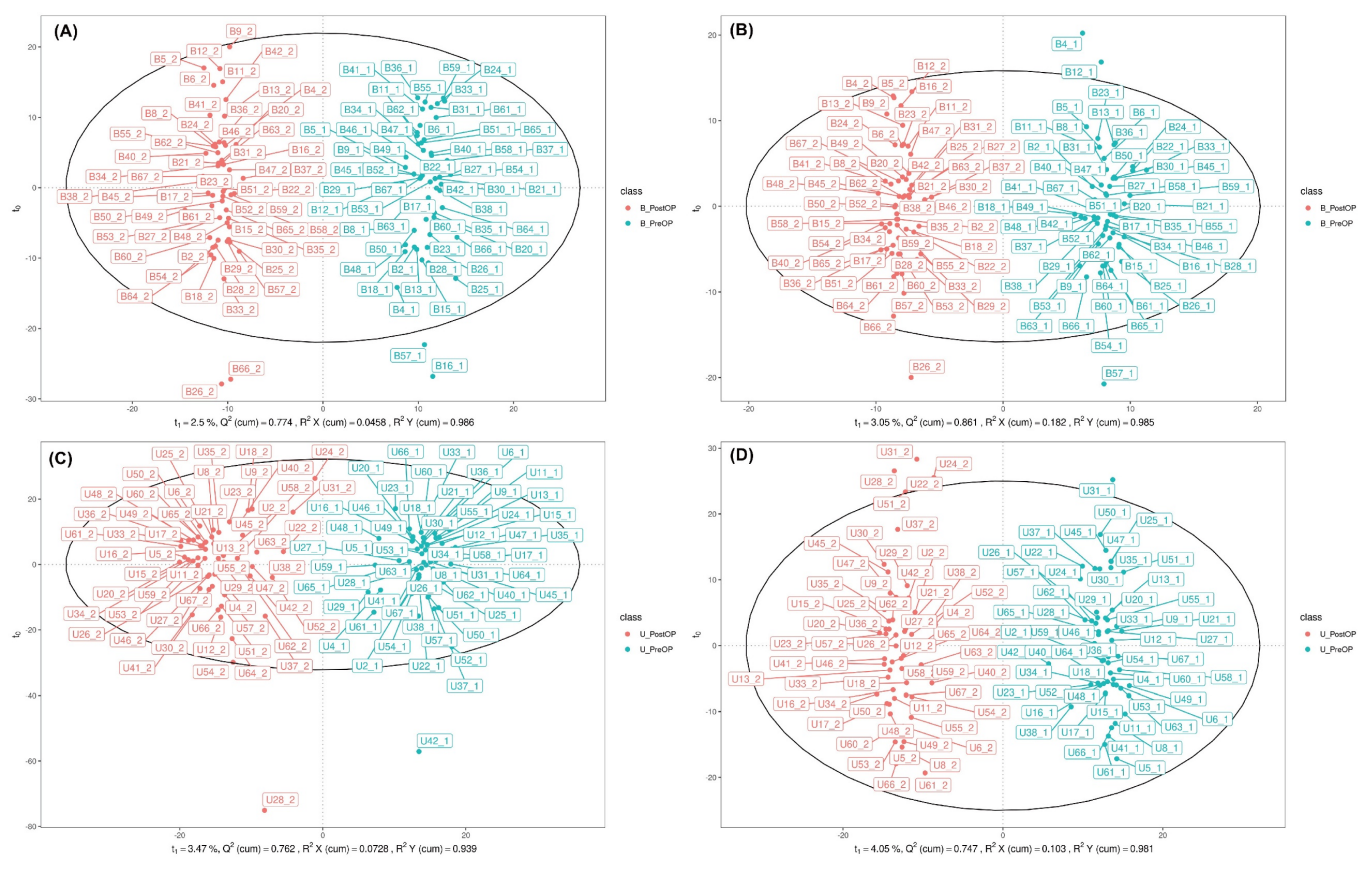
Orthogonal partial least squares discriminant analysis of perioperative plasma and urine metabolomics. A. (ESI+) metabolites in plasma. B. (ESI-) metabolites in plasma. C. (ESI+) metabolites in urine. D. (ESI-) metabolites in urine. B_PreOP - metabolites identified preoperatively in blood (plasma). B_PostOP - metabolites identified postoperatively in blood (plasma). U_PreOP - metabolites identified preoperatively in urine. U_PostOP - metabolites identified postoperatively in urine.

In total, 4762 molecular features of plasma and 6190 molecular features of urine in ESI positive (ESI+) mode and 2190 molecular features of plasma and 4171 molecular features of urine in ESI negative (ESI-) mode were obtained. Overall, 191 endogenous metabolites in plasma and 266 endogenous metabolites in urine, defined by certain MS derived signals based on chromatographic retention time and accurate mass, were identified. After permutation correction for multiple measurements, significant changes in 27 metabolites in plasma and 32 metabolites in urine were observed. To visualize the metabolic perturbations induced by the surgical procedure, OPLS-DA models were constructed for both blood plasma and urine samples, analyzed in both positive (ESI+) and negative (ESI-) ionization modes (Figure 2).

The OPLS-DA scores plots for blood plasma (Figures 2A and 2B) demonstrated a clear and distinct separation between the pre-operative (PreOP) and post-operative (PostOP) groups. For the ESI+ mode data (Figure 2A), the model showed excellent performance with high goodness-of-fit (R^2^Y = 0.996) and strong predictive capability (Q^2^ = 0.898), indicating a robust metabolic distinction between the two time points. Similarly, the model for the ESI- mode in blood (Figure 2B) also revealed a significant separation, supported by strong validation metrics (R^2^Y = 0.985, Q^2^ = 0.865). Consistent results were observed in the analysis of urine samples (Figures 2C and 2D). The OPLS-DA model for urinary metabolites in ESI+ mode (Figure 2C) successfully discriminated between the PreOP and PostOP states, yielding a model with high predictive power (R^2^Y = 0.939, Q^2^ = 0.828). Likewise, the analysis of ESI- mode urinary metabolites (Figure 2D) resulted in a clear separation pattern with robust model parameters (R^2^Y = 0.983, Q^2^ = 0.933). Collectively, the distinct clustering observed in all four OPLS-DA models strongly suggests that the surgical intervention induced significant and detectable alterations in the systemic metabolic profiles of both blood plasma and urine.

**Figure 3:**
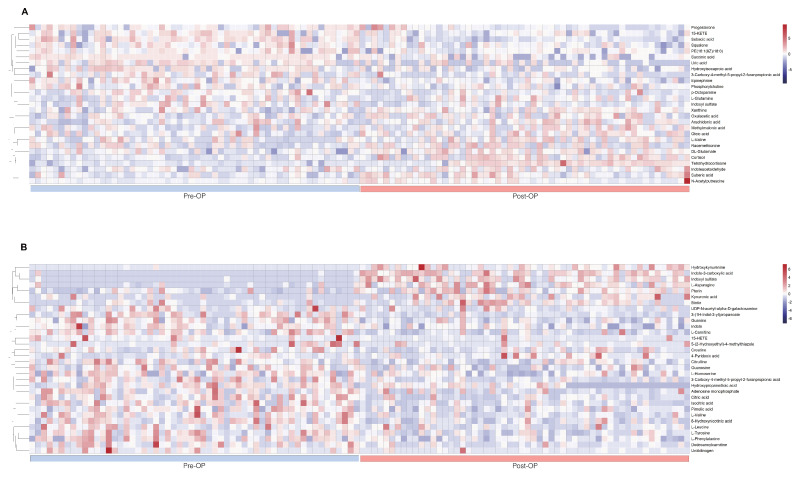
Perioperative heat maps representing hierarchical clustering of metabolites in plasma and urine during the lumbar spinal surgery. The row displays data for a specific metabolite, and each column shows the samples. Red and blue denote high and low relative concentrations, respectively. A. Heat map displays the preoperative and postoperative clustering of plasma metabolites (n= 27). B. Heat map displays the preoperative and postoperative clustering of urinary metabolites (n= 32).

The Table 4 summarized intraoperative changes in metabolites in terms of changes in multiple metabolic pathways, specifically steroid hormone synthesis (n = 4 in the plasma sample), protein catabolism (n = 7 in the plasma sample, n = 16 in the urine sample), glucose metabolism (n = 3 in the plasma sample, n = 1 in the urine sample), catecholamine (n = 2 in the plasma sample), phospholipid breakdown (n = 4 in the plasma sample, n = 1 in the urine sample), fatty acid metabolism (n = 4 in the plasma sample), and purine and pyrimidine metabolism (n = 2 in the plasma sample, n = 4 in the urine sample). Metabolites of protein catabolism were the most commonly observed and included glucogenic amino acids, metabolites of muscle wasting, and tryptophan degradation. These intraoperative metabolic variations were presented on heat maps, as are results of hierarchical cluster analysis (Fig. 3) for intuitive visualization.

**Figure 4:**
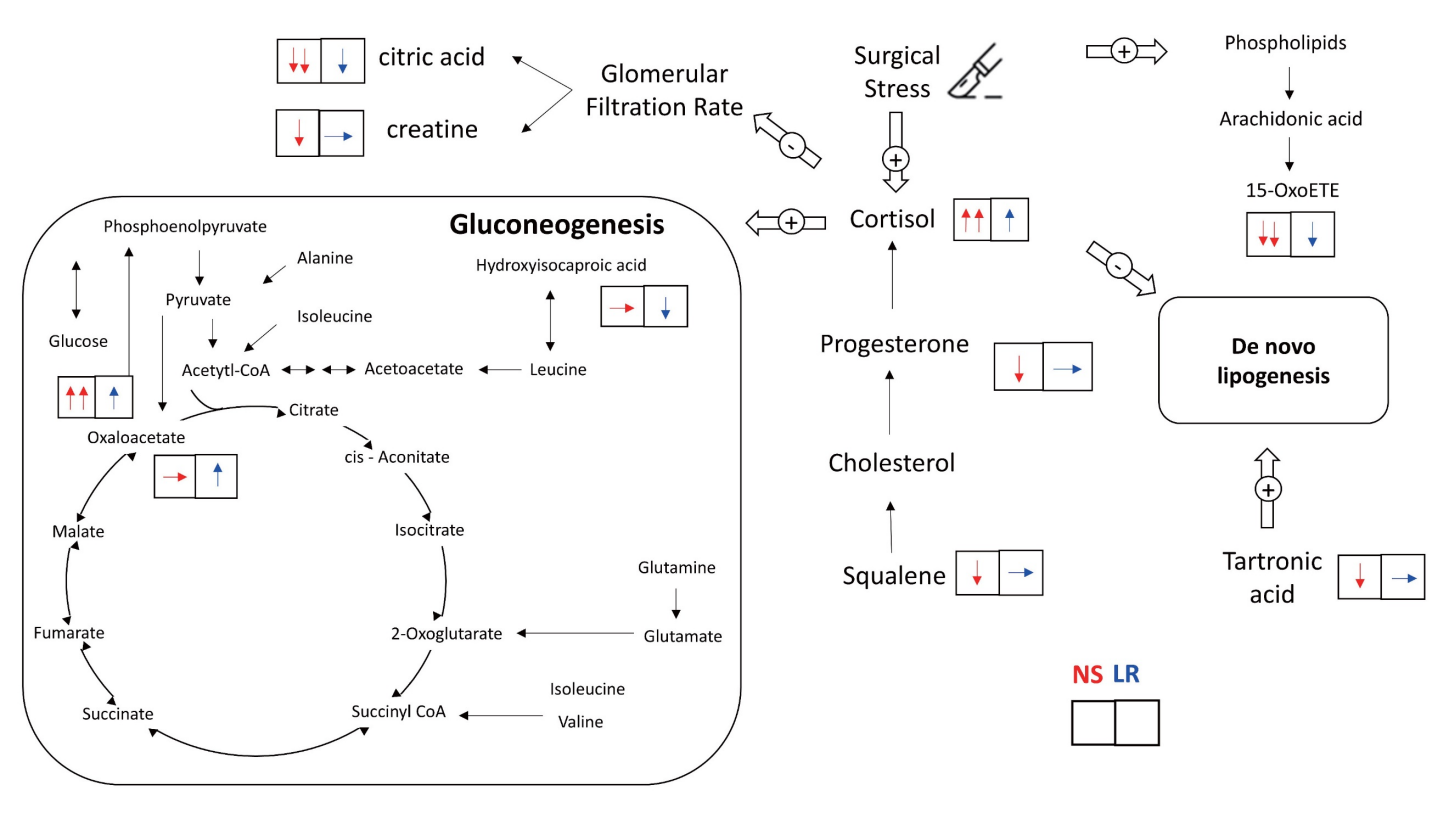
Intraoperative metabolic alterations from administration of 0.9% saline and lactated Ringer's solutions. The upward arrow (↑) indicates a significant increase in the metabolite level after the surgery and the downward arrow (↓) indicates a significant decrease in the metabolite level after the surgery. The flat arrow (→) indicates no significant change in the metabolite level during the surgery.

The effects of the type of crystalloid solution on these intraoperative metabolic pathways are summarized in Figure 4. Patients receiving 0.9% saline solution had strong trends of higher plasma cortisol concentrations (interaction *P* = .051) and lower plasma cortisol precursor concentrations, including progesterone (interaction *P* = .061) and squalene (interaction *P* = .099), after surgery than patients receiving lactated Ringer's solution. By comparison, patients receiving lactated Ringer's solution but not those receiving 0.9% saline solution exhibited a significant increase in plasma oxaloacetate concentrations, which may indicate reduced gluconeogenesis (interaction *P* = .015). Patients receiving lactated Ringer's solution also had a strong trend of lower concentrations of plasma hydroxyisocaproic acid (interaction *P* = .055), a leucine degradation metabolite, and smaller decreases in phospholipid breakdown anti-inflammatory metabolite concentrations, namely 15-ketoeicosatetraenoic acid (15-oxoETE, interaction *P* = .063). With regard to urinary metabolomic profiles, patients receiving 0.9% saline solution revealed strong trends of reduced excretion of citric acid (interaction *P* = .083) and creatine (interaction *P* = .079), both metabolites are in relation to a lower glomerular filtration rate, and a strong trend of lower concentration of hydroxypropanedioic acid (interaction *P* = .059), which is associated with de novo lipogenesis.

## Discussion

Previous applications of metabolomic analysis in a surgical population have primarily focused on its diagnostic and prognostic properties 8; the influence of perioperative interventions has not been thoroughly explored. Recently, Seymour et al. investigated acute perioperative alterations in plasma and urine metabolomic profiles among patients undergoing elective cardiovascular, gastrointestinal, hernia, oncologic, and urologic surgeries 11. They reported that elective surgery induces broad, early, and measurable metabolic changes, including lipolysis, glycolysis, and amino acid oxidation, along with increased excretion of gut microbe-derived toxins 11. Using a similar metabolomic approach, the present study aimed to further elucidate the physiological characteristics of lumbar spinal surgery and to explore the effects of intravenous crystalloid infusion. We observed that lumbar spinal surgery induced several major metabolic pathway alterations, such as steroid hormone synthesis (promoting cortisol production), glucose metabolism (facilitating gluconeogenesis), protein catabolism (elevation of plasma glucogenic amino acids and muscle wasting), and breakdown of phospholipids (arachidonic acid release and associated metabolite consumption in plasma). Furthermore, we observed that the 0.9% saline and lactated Ringer's solutions differ in how they affect a few key intraoperative metabolic pathways.

Our LC-MS findings indicated that plasma cortisol concentrations were higher in patients receiving 0.9% saline solution than in patients receiving lactated Ringer's solution. Perioperative changes in plasma cortisol concentrations have been extensively investigated in the literature, but LC-MS, which is the gold standard of cortisol measurement, has been applied in fewer than 3% of studies, and no cortisol measurements made using LC-MS has been undertaken during or immediately after surgery 27. In addition, we observed that precursor concentrations of cortisol, namely progesterone and squalene 28, were lower in patients receiving 0.9% saline solution than in those receiving lactated Ringer's solution. This is consistent with our finding of the difference in plasma cortisol concentrations. Because cortisol is a potent trigger of gluconeogenesis 29, we consistently observed that patients receiving 0.9% saline solution also had higher intraoperative plasma glucose concentrations. Some other possible mechanisms affecting the difference in glucose concentrations between the two groups are as follows. First, patients receiving 0.9% saline solution presented with hypernatremia, which is associated with increased plasma cortisol concentrations 30. Second, serum pH is lower in patients receiving 0.9% saline solution than in those receiving lactated Ringer's solution. A lower serum pH facilitates renal gluconeogenesis, which leads to higher serum glucose concentrations 31. Third, the lactate of the lactated Ringer's solution may act as a bioenergetic fuel substitute for oxaloacetate in surgery-induced gluconeogenesis, thereby preventing the consumption of oxaloacetate. Accordingly, we observed that plasma oxaloacetate concentrations were higher in patients receiving lactated Ringer's solution than 0.9% saline solution. Fourth, chloride interacts with many protein kinases, including cyclic AMP (cAMP) protein kinases 32, and both gluconeogenesis 33 and steroid hormone production 34 are regulated by cAMP protein kinases. Therefore, these two metabolic pathways may be altered by 0.9% saline solution-induced hyperchloremia. Furthermore, we observed that patients receiving 0.9% saline required significantly higher norepinephrine doses compared with those receiving lactated Ringer's solution. This finding is consistent with a relevant randomized controlled trial that compared 0.9% saline with balanced crystalloids in major abdominal surgery 35. The underlying mechanism may be related to the vasodilatory effects of elevated serum chloride, in addition to its influence on intraoperative cortisol synthesis 36.

Cortisol inhibits de novo lipogenesis 37 and participates in intraoperative protein breakdown 4, 29, 38. We observed that urinary hydroxypropanedioic acid concentrations (tartronic acid) significantly decreased in patients receiving 0.9% saline solution but did not decrease in patients receiving lactated Ringer's solution. Tartronic acid promotes de novo lipogenesis 39, and the dysregulation of de novo lipogenesis is associated with insulin resistance and the perturbation of glucose metabolism 37. This result indicated unfavourable effects of 0.9% saline infusion versus the lactated Ringer's solution. In the present study, we also observed substantial amounts of metabolites of protein catabolism in both plasma and urine samples. For instance, changes in glucogenic amino acid concentrations, including those of branched-chain amino acids (BCAAs), and tryptophan catabolic metabolites were observed after surgery. Furthermore, we observed lower plasma concentrations of leucine degradation metabolites, namely hydroxyisocaproic acid (HICA), in patients receiving lactated Ringer's solution than in those receiving 0.9% saline solution. Leucine, a BCAA, is consumed in the liver, muscle, and adipose tissue for protein synthesis and glucose homeostasis. Intraoperative protein breakdown, particularly the appearance of leucine in plasma, is associated with glucose concentrations 38 and elevated plasma HICA concentrations are associated with a breakdown of protein for energy 40. Therefore, the lower plasma HICA concentrations in patients receiving lactated Ringer's solution may indicate a decrease in intraoperative protein breakdown due to a smaller increase in intraoperative cortisol. The mitigation of surgical stress, maintenance of intraoperative glucose homeostasis, and prevention of protein breakdown are encouraged as part of surgical recovery protocols 4, 41. A balanced crystalloid solution is recommended as the intraoperative intravenous solution versus 0.9% saline solution.

When cells are stressed, arachidonic acid is released from phospholipids and then oxidized or modified into various bioactive metabolites that regulate the inflammatory response by promoting and inhibiting the inflammatory cascade 42. In the present study, we observed surgical stress-induced increases in plasma arachidonic acid concentrations and decreases in several arachidonic acid metabolites in both plasma and urine samples. In particular, we observed a more prominent decrease in 15-oxoETE, an arachidonic metabolite with anti-inflammatory properties 42, among patients receiving 0.9% saline solution than among those receiving lactated Ringer's solution. This is consistent with a previous study that found that a balanced solution was less likely to induce an inflammatory response during major abdominal surgery than an unbalanced solution 43.

With regard to the influence of crystalloid solution on renal metabolism, our findings are consistent with those of previous studies indicating that 0.9% saline solution is associated with a higher risk of perioperative acute kidney injury 1. These findings may be associated with higher plasma chloride and cortisol concentrations. Plasma chloride homeostasis is mainly regulated by the kidney, and hyperchloremia induces renal vasoconstriction of the vas afferens and a decrease in the glomerular filtration rate 32. In addition, a perioperative increase in cortisol may contribute to renal injury by compromising renal perfusion 27. We observed that renal excretion of creatine, which correlated with the glomerular filtration rate 44, were lower in patients receiving 0.9% saline solution with accompanying hyperchloremia. Furthermore, urinary citrate excretion decreases are associated with serum glucose increases and glomerular filtration rate decreases 45. We compatibly observed that patients receiving 0.9% saline solution had significantly lower urinary citrate excretion and higher intraoperative serum glucose than patients receiving lactated Ringer's solution. To our knowledge, no previous randomized controlled trial has investigated the renal metabolomic effects of intraoperative fluid infusion. Kidney transplant recipients are the most susceptible surgical population to crystalloid choice, and our observed metabolomic differences may provide mechanistic insight into the previously reported benefit of balanced crystalloids in reducing delayed graft function compared with 0.9% saline 7.

### Limitations

The present study has several limitations. First, it focused on intraoperative metabolism. Postoperative metabolism is theoretically influenced by more factors and was not investigated in the current study because the variation in the effects of intraoperative crystalloid solutions may be masked. Second, we set up strict inclusion criteria to minimize the confounding effects of preoperative comorbidities on intraoperative metabolism. Patients with comorbidities may present different metabolic profiles. Third, untargeted metabolomic analysis yields relative metabolite concentrations instead of absolute concentrations; future studies should adopt targeted metabolomic analysis to increase the accuracy of metabolite identification. The present study may be useful as reference for future research using targeted metabolomic analysis 20.

In conclusion, this study demonstrates that the administration of lactated Ringer's solution to middle-aged patients undergoing lumbar spinal surgery elicited more favorable intraoperative catabolic and metabolic pathways implicated in cortisol synthesis, glucose homeostasis, and metabolites in relation to glomerular filtration rate when compared to 0.9% saline solution.

## Figures and Tables

**Table 1:** Patient characteristics

	NS group (n= 28)	LR group (n= 28)	Standardized difference
Age (yr)	55.9 ± 7.0	52.7 ± 6.6	0.46
Weight (kg)	69.6 ± 11.5	67.0 ± 9.0	0.25
Height (cm)	160.4 ± 8.3	162.8 ± 7.8	0.30
Female sex (n;%)	15 (54.6%)	15 (54.6%)	0
Comorbidity (n; %)			
Hypertension	14 (50.0%)	11 (39.3%)	0.22
Diabetes (no insulin use)	6 (21.4%)	4 (14.3%)	0.19
Smoker	4 (14.3%)	4 (14.3%)	0
ASA class (n; %)			
I	22 (78.6%)	21(75.0%)	0.09
II	6 (21.4%))	7 (25.0%)	0.09
Type of surgery (n; %)			
Decompression	11 (39.3%)	12 (42.8%)	0.07
Fusion	16 (57.1%)	15 (53.6%)	0.07
Benign tumor resection	1 (3.6%)	1 (3.6%)	0

Data are presented as mean (standard deviation), number, and percentage as appropriate. A standardized difference >0.52 indicates imbalance between the groups.

**Table 2:** Intraoperative profiles and postoperative outcomes

	NS group (n= 28)	LR group (n= 28)	*P* value
Anesthesia time (min)	202 ± 72	188 ± 55	0.403
Blood loss (ml)	40 (30-200)	40 (30-110)	0.638
Fentanyl dose (mcg)	154 ± 67	154 ± 47	1.000
Crystalloid amount (ml)	1706 ± 522	1588 ± 511	0.394
Urine output (ml)	224 ± 163	284 ± 136	0.165
Norepinephrine dose (μg)	18 (0-43)	0 (0-5)	< 0.001
Complication (n; %)			0.406
Infection	1 (3.6%)	0 (0%)	
Arrythmia	0 (0%)	2 (7.2%)	
New neuro symptom	1 (3.6%)	1 (3.6%)	
Rebleed	1 (3.6%)	0 (0%)	
Length of hospital stay (d)	5 (4-6.5)	4 (3-5)	0.235

Data are presented as mean (standard deviation), median (interquartile range), number, and percentage as appropriate.

**Table 3:** Intraoperative arterial blood gas profile

	Group	Baseline (mean ± SD)	End of surgery (mean ± SD)	Change (End-Baseline), mean ± SD	ΔΔ (NS-LR), mean (95% CI)	Interaction P
pH	NS	7.404 ± 0.040	7.346 ± 0.040	-0.057 ± 0.036	**-0.024** (-0.047 to -0.002)	0.034
	LR	7.424 ± 0.040	7.391 ± 0.050	-0.033 ± 0.046		
Sodium (mmol/L)	NS	137.533 ± 1.517	138.164 ± 2.296	+0.632 ± 1.357	**+1.389** (0.372 to 2.407)	0.008
	LR	137.318 ± 1.877	136.561 ± 1.640	-0.757 ± 1.397		
Chloride (mmol/L)	NS	108.071 ± 2.613	110.643 ± 2.270	+2.571 ± 2.332	**+1.607** (0.582 to 2.633)	0.003
	LR	106.464 ± 2.066	107.429 ± 2.308	+0.964 ± 1.374		
Glucose (mg/dL)	NS	100.893 ± 22.987	125.214 ± 24.817	+24.321 ± 22.719	**+11.179** (0.495 to 21.862)	0.041
	LR	102.321 ± 17.775	115.464 ± 27.884	+13.143 ± 25.302		

ΔΔ means the difference in time-differences between the two study groups

**Table 4:** Variations in intraoperative metabolites

Plasma (27 metabolites)	Direction of change (Fold change)	P value
Steroid hormone synthesis (4 metabolites)		
Cortisol	↑(1.957)	< 0.001
Tetrahydrocortisone	↑(1.910)	< 0.001
Progesterone	↓ (0.813)	0.04
Squalene	↓ (0.847)	0.004
Protein catabolism (7 metabolites)		
Glutamine (glucogenic)	↑ (1.137)	0.037
Glutamate (glucogenic)	↑ (1.272)	<0.001
Valine (BCAA; glucogenic)	↑ (1.454)	0.004
Methionine (glucogenic)	↑ (1.397)	0.041
Hydroxyisocaproic acid (leucine metabolite)	↓ (0.774)	0.007
Indoxyl sulfate (tryptophan metabolite)	↓ (0.646)	< 0.001
Indoleacetaldehyde (tryptophan metabolite)	↑ (1.176)	0.003
Glucose metabolism (3 metabolites)		
Succinic acid	↓ (0.651)	< 0.001
Oxalacetate	↑ (1.298)	0.018
Methylmalonic acid	↑ (1.411)	0.01
Catecholamine (2 metabolites)		
Octopamine	↓ (0.561)	< 0.001
Epinephrine	↓ (0.708)	< 0.001
Phsopholipid breakdown (4 metabolites)		
Arachidonic acid	↑ (1.237)	0.001
15-oxoETE	↓ (0.741)	< 0.001
Phosphorylcholine	↓ (0.894)	0.04
PE(16:1(9Z)/18:0)	↓ (0.864)	0.049
Fatty acid metabolism (4 metabolites)		
Sebacic acid	↓ (0.528)	< 0.001
Oleic acid	↑ (1.448)	0.003
Suberic acid	↑ (1.616)	< 0.001
3-Carboxy-4-methyl-5-propyl-2-furanpropionic acid	↓ (0.421)	< 0.001
Purine & pyramidine metabolism (2 metabolites)		
Uric acid	↓ (0.496)	< 0.001
Xanthine	↑ (0.231)	0.005
Others (one metabolite)		
N-Acetylputrescine (polyamine)	↑ (3.09)	< 0.001
Group by time interaction effect in plasma (6 metabolites)		Interaction P value
Cortisol	NS ↑↑; LR↑	0.0510
Progesterone	NS↓; LR-	0.06090
Squalene	NS↓; LR-	0.0991
Oxaloacetate	NS-; LR↑	0.0149
15-OxoETE	NS↓↓; LR↓	0.0625
Hydroxyisocaproic acid	NS-; LR↓	0.0554
Urine (32 metabolites)	Direction of change (Fold change)	P value
Protein catabolism (16 metabolites)		
3-(1H-Indol-3-yl)propanoate (tryptophan metabolite)	↓(0.298)	< 0.001
Indole-3-carboxylic acid (tryptophan metabolite)	↑(22.356)	< 0.002
Indoxyl sulfate (tryptophan metabolite)	↑(36.461)	< 0.001
Indole (tryptophan metabolite)	↓(0.723)	< 0.001
Hydroxykynurenine (tryptophan metabolite)	↑(23.695)	< 0.001
Kynurenic acid (tryptophan metabolite)	↑(3.205)	< 0.001
Carnitine (muscle wasting)	↓(0.489)	< 0.001
Creatine (muscle wasting)	↓(0.667)	0.002
Dodecanoylcarnitine (muscle wasting)	↓ (0.420)	< 0.001
Tyrosine (Glucogenic)	↓(0.588)	< 0.001
Asparagine (Glucogenic)	↑(4.210)	< 0.001
Leucine (BCAA; Glucogenic)	↓(0.722)	0.017
Valine (BCAA; Glucogenic)	↓(0.415)	< 0.001
Citrulline	↓(0.567)	< 0.001
Homoserine	↓ (0.685)	< 0.001
Phenylalanine	↓ (0.600)	< 0.001
Glucose metabolism (one metabolite)		
Hydroxypropanedioic acid	↓(0.590)	< 0.001
Phospholipid breakdown (one metabolite)		
15-Hydroxyeicosatetraenoic acid	↓(0.397)	< 0.001
Purine & pyramidine metabolism (4 metabolites)		
Guanine	↓(0.649)	< 0.001
Guanosine	↓(0.734)	0.009
Adenosine monophosphate	↓(0.546)	< 0.001
UDP-N-acetyl-alpha-D-galactosamine Citric acid cycle (2 metabolites)	↓ (0.749)	0.019
Isocitric acid	↓ (0.433)	< 0.001
Citric acid	↓ (0.466)	< 0.001
Others (8 metabolites)		
Pterin	↑ (1.993)	< 0.001
Pimelic acid (vitamin B7 synthesis)	↓ (0.653)	< 0.001
4-Pyridoxic acid (vitamin B6 catabolism)	↑ (1.796)	0.013
6-Hydroxynicotinic acid	↓ (0.553)	< 0.001
Biotin (vitamin B7)	↑ (2.634)	< 0.001
3-Carboxy-4-methyl-5-propyl-2-furanpropionic acid (uremic toxin)	↓ (0.426)	< 0.001
Urobilinogen (bile acid metabolite)	↓(0.362)	0.001
5-(2-Hydroxyethyl)-4-methylthiazole	↓(0.459)	0.031
Group by time interaction effect in urine (3 metabolites)		Interaction P value
Citric acid	NS↓↓; LR↓	0.0831
Creatine	NS↓; LR-	0.0794
Hydroxypropanedioic acid	NS↓; LR-	0.0586

BCAA: branched-chain amino acid, 9,10-DHOME: 9,10-dihydroxy-12-octadecenoic acid, UDP: uridine diphosphate ↑means an increase after surgery with a P value< 0.05 and ↓means a decrease after surgery with a P value< 0.05.
